# Directed growth of quinacridone chains on the vicinal Ag(35 1 1) surface

**DOI:** 10.3762/bjnano.15.48

**Published:** 2024-05-21

**Authors:** Niklas Humberg, Lukas Grönwoldt, Moritz Sokolowski

**Affiliations:** 1 Clausius-Institut für Physikalische und Theoretische Chemie der Universität Bonn, Wegelerstrasse 12, 53115 Bonn, Germany

**Keywords:** Ag(100), intermolecular hydrogen bonds, one-dimensional aggregates, organic nanostructures, quinacridone, step-molecule interactions, vicinal surface

## Abstract

The formation of self-assembled domains and chains of monomolecular width of quinacridone (QA) on the vicinal Ag(35 1 1) surface was investigated by scanning tunneling microscopy and low-energy electron diffraction. The focus was on the influence of the steps on the QA structures and their preferential azimuthal orientations with the aim of achieving a selective orientation. After deposition at a sample temperature of 300 K, QA forms the same kind of molecular chains as on the nominally flat Ag(100) surface because of strong intermolecular hydrogen bonds, which we reported in a previous publication [Humberg, N.; Bretel, R.; Eslam, A.; Le Moal, E.; Sokolowski, M. *J. Phys. Chem. C* **2020,** *124,* 24861–24873]. The vicinal surface leads to one additional chain orientation, which is parallel to the Ag step edges. However, most chains nucleate on the Ag terraces between steps with four distinct azimuthal orientations that are identical to those on Ag(100), and which are determined by the interactions with the (100) surface. At 300 K, the chains grow across the Ag steps, which do not break the azimuthal chain orientations. In contrast, during the deposition at sample temperatures of 400 and 500 K, the nucleation of the chains takes place at the Ag step edges. Hence, these have a strong influence on the azimuthal orientation of the molecules, resulting in a preferential growth of the chains in two of the four azimuthal orientations. We explain this by the adaptation of favorable adsorption sites, which involve the replacement of Ag atoms by QA molecules with specific azimuthal orientations at the step edges.

## Introduction

A versatile and powerful method to create nanostructures on surfaces is the self-assembly of atoms and molecules. Here, the physical and chemical properties of the substrate and the adsorbate are the key that can be tuned to create nanostructures that fit specific needs [1–4]. A research focus over the recent years has been the self-assembly of molecules or atoms into one-dimensional (1D) linear aggregates. They allow for electron transport along the long axes of the 1D aggregates, while a confinement effect is present along their short axes. Hence, they are considered as building blocks for new generations of devices for computing, photovoltaics, thermoelectrics, and energy storage [5–7]. Furthermore, one-dimensional aggregates are also used for gas sensing and carbon-capturing materials [8–9].

A group of surfaces that is very appealing for the growth of 1D structures are vicinal surfaces [10] because the step edges break the rotational symmetry of the surface further and add a periodic 1D grating, which can be used to direct the growth of nanostructures of adsorbates. The adsorption at step edges, as opposed to that on the terraces in between, is often favored because the additional interactions between the adsorbate and the atoms of the step edge contribute to the adsorption energy *E*_ads_. This step decoration can be exploited to grow 1D chain-like structures of adsorbates that otherwise tend to form two-dimensional (2D) domains on flat terraces. Examples are atoms forming 1D metallic chains [11–15] and organic molecules forming 1D chains at step edges of vicinal metal surfaces at a low coverage, for example, PTCDA on Au(433) and Au(778) [16], a 1:1 mixture of PTCDI and 1,4-bis(2,4-diamino-1,3,5-triazine)benzene on Au(11 11 12) [17], and nickel-tetraphenyl-porphyrin on Au (788) [18].

Here, we report on the growth of an organic molecule, namely 5,12-dihydroquino[2,3-*b*]acridine-7,14-dione (quinacridone, QA), which forms 1D chains on flat metal surfaces already per se, on a vicinal Ag(100) surface. We describe how the presence of the step edges influences the azimuthal orientations of the chains. QA is a good candidate for such experiments because strong intermolecular hydrogen bonds (H-bonds) [19] support the growth of long 1D chains of parallel oriented QA molecules, both in bulk crystals and on surfaces.

**Figure 1:**
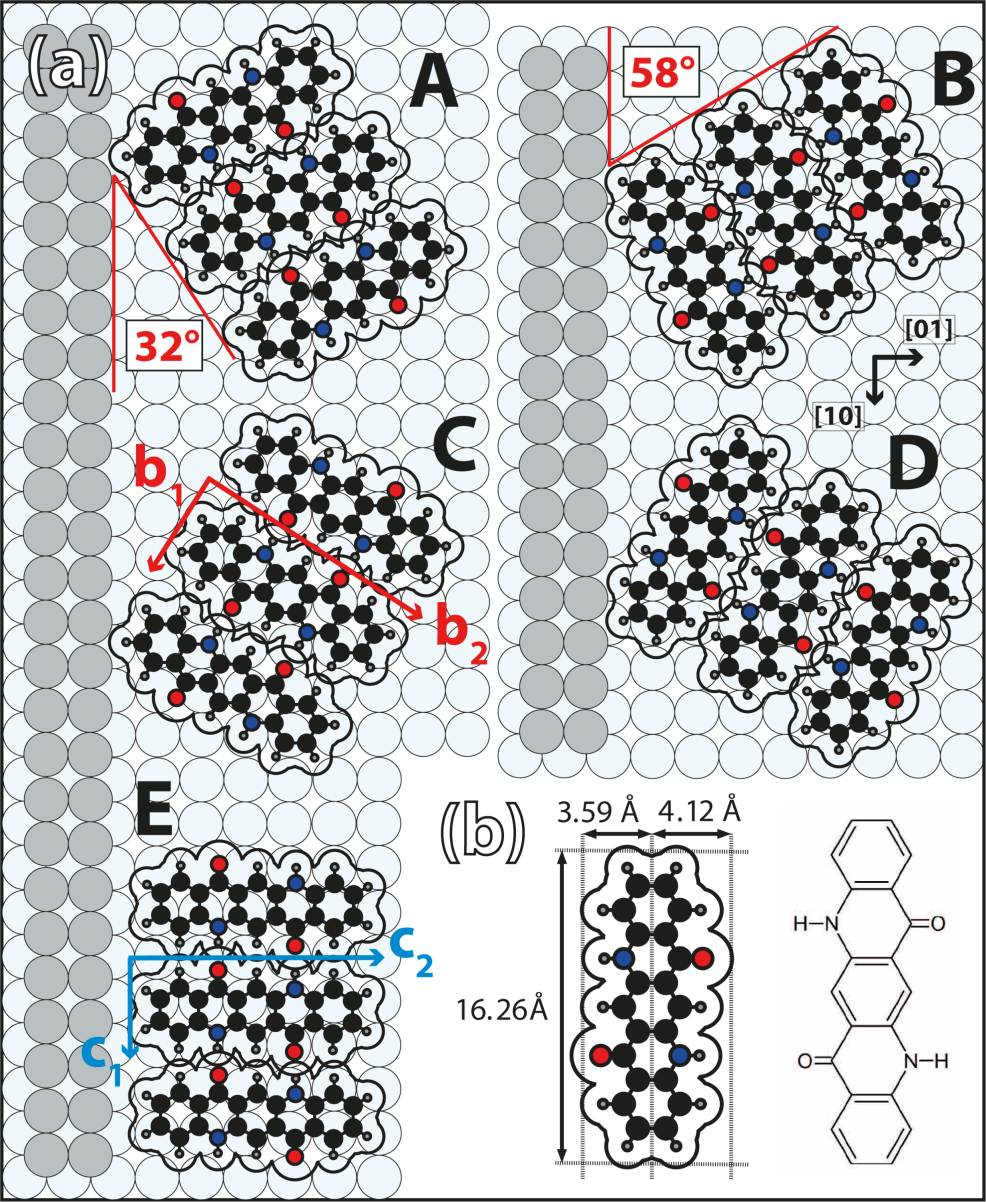
(a) Models of the five observed azimuthal QA chain orientations, A–E, relative to the unidirectional step edges of the Ag(35 1 1) surface. The four orientations A–D are identical to those that were already observed on the nominally flat Ag(100) surface [23]. The chain directions A and C, as well as B and D, exhibit equivalent adsorption sites because of a mirror symmetry. Note that the adsorbed molecules in A(C) and B(D) are of the same chirality R(L). For the chain orientation E there exists the energetically equivalent chain with molecules of the opposite chirality. The color code of the atoms is: black = carbon, gray = hydrogen, blue = nitrogen, and red = oxygen. (b) Structural formula and hard-sphere model with dimensions of QA. The nominal van der Waals radii used for the molecular models were taken from [27].

It has been shown by Głowacki et al. that QA exhibits promising properties for applications in electronic and optoelectronic devices [20–21]. In particular, they found a hole mobility of 0.1 cm^2^·V^−1^·s^−1^ and a photocurrent of about 1 mA·cm^−2^. These observations were both attributed to a close packing of the molecules due to strong intermolecular H-bonds. The empirical formula of QA is C_20_H_12_N_2_O_2_, and a structural formula and a hard-sphere model of QA are displayed in Figure 1b. The self-assembly of QA has already been investigated on some nominally flat surfaces such as Ag(111) [22], Ag(100) and Cu(111) [23], and graphene and MoS_2_ [24–25]. These studies have shown that QA grows in long one-dimensional chains connected by H-bonds. The chains exhibit a small set of distinct orientations, which are determined by the underlying substrates on all these surfaces. Furthermore, chains of QA have also been observed on insulating layers of KCl grown on Ag(100) or Cu(111) [19,26].

In this work, we report the growth of QA on the vicinal Ag(35 1 1) surface. The central question of this work was whether the unidirectional step edges influence the azimuthal orientations of the QA chains with respect to those that were observed on the nominally flat Ag(100) surface [23]. We found that the azimuthal orientations are identical to those on Ag(100), but, at elevated temperatures, the periodic step edges lead to a preferential growth of two distinct orientations out of four. Furthermore, we observed the growth of one, hitherto not described, azimuthal chain orientation parallel to the Ag step edges.

## Experimental

The experiments were conducted in an ultrahigh vacuum chamber with a base pressure of 2 × 10^−10^ mbar equipped with a beetle-type scanning tunneling microscope (STM, type UHV 300) from RHK Technology, a microchannel plate low-energy diffraction (MCP-LEED) instrument from OCI Vacuum Microenginneering Inc., and a quadrupole mass spectrometer (QMS) of the type PRISMA from Pfeiffer Vacuum. The vicinal Ag(35 1 1) crystal was obtained from MaTecK and cleaned by repeated cycles of sputtering and subsequent annealing at 700 K for 45 min. We tested whether a variation of the annealing temperatures between 500 and 800 K has an influence on the step distribution of the surface. However, no significant influence on the step distribution was observed. The purified QA [28] was evaporated from a custom-built evaporator at a crucible temperature of 720 K, while the sample was held at different temperatures between 300 and 500 K in order to test the role of the growth temperature. The deposition process was monitored by the QMS, and the integrated QMS signal was used to calculate the QA coverage θ_QA_. It is given in numbers of monolayers (ML) of the α-phase, as explained in detail in [23]. A more detailed description of the experimental procedures can be found in [23].

The LEED measurements were performed at 300 K with beam currents below 10 nA and electron energies (*E*) between 30 and 200 eV. Note that the reported diffraction patterns are distorted due to the MCP geometry. All STM images were recorded at room temperature. As a tip, we used a self-cut Pt/Ir (90:10) wire. The bias voltage (*U*_Bias_) refers to the sample, and the tunneling current (*I*) was at a constant value in the range of 10–50 pA. We usually adjusted the scanning plane parallel to the (35 1 1) plane of our sample in order to optimize the contrast of the images. The images were processed with the program SPIP [29], which included filtering out noise and an adjustment of the contrast. Additionally, to some images, a Prewitt or Roberts filter for edge enhancement was applied in order to enhance the visibility of the step edges.

The structure models were drawn with the free software Graphics Layout Engine [30]. The shown hard-sphere models of QA use bond lengths [31] and van der Waals radii [27] from the literature.

## Results and Discussion

### The Ag(35 1 1) surface

The Ag(35 1 1) surface is vicinal (i.e., tilted by an angle of 2.3°) with respect to the Ag(100) surface. The step direction is along the [10] direction. In this work, we use the 2D unit cell of the Ag(100) terraces for reference. The corresponding [10] and [01] unit cell vectors are illustrated in Figure 1. These two orientations correspond to the [011] and the 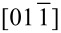 directions of the bulk crystal structure, respectively. In the LEED images (reciprocal space), the labels [10] and [01] also refer to the corresponding directions in real space and those of the reciprocal unit cell vectors.

On average, the steps are separated by a length of Λ = 50.5 Å or 17.5 atom rows. Regarding possible QA chains (see, e.g., Figure 1) on this surface, this means that up to three chains, parallel to an adjacent step edge, would fit on a terrace (one chain is 16.5 Å in width). A QA chain that is oriented perpendicular to the step edges would consist of seven molecules on one terrace (the intermolecular distance within a QA chain is 6.6 Å).

An STM image of the Ag(35 1 1) surface is shown in Figure S1a of Supporting Information File 1. The STM image reveals that the Ag steps are not regularly spaced. Instead, the distribution of the terrace widths is very broad. The step distribution that was obtained by evaluating STM images with an Python script reported by Bastidas et al. [32] is shown in Figure S1b in Supporting Information File 1. On the left-hand side of the STM image (Figure S1, Supporting Information File 1), the average distance between the steps is only 20 Å (seven atom rows), which is less than half of the expected terrace width of 50.5 Å. At the same time, the surface also exhibits flat areas with wide terraces of up to 300 Å in width. This can also be seen in the STM image. A similar type of step width distribution was also found for various other surfaces, for example, Cu(11*n*) [33] with *n* = 5, 9, 17, and Si(100) as well as Si(111) [34]. These aspects are of importance for the formation of QA structures on Ag(35 1 1), which will be reported below.

### Deposition at 300 K

In many aspects, the growth of QA on Ag(35 1 1) is very similar to its growth on the nominally flat Ag(100) surface [23]. Upon deposition at a sample temperature of 300 K, QA forms the same kind of parallel chains of molecules that are connected via H-bonds, which can only be formed between molecules of the same handedness [23] on the surface. Hence, the chains are homochiral, and chains of the same azimuthal orientation consist of the same enantiomers. Like on Ag(100), the distance between the chains, *b*_2_, depends on the coverage as they repel one another because of a substrate-mediated interaction. The intermolecular distance along the chains is *b*_1_ = 6.6 ± 0.2 Å, and is, within the margins of error, identical to the distance on Ag(100) [23] (see Figure 1). The chains exhibit four distinct azimuthal orientations, which are identical to the ones that we observed previously on the nominally flat Ag(100) surface [23] and will be named A–D in the following. Models of the four chain directions A–D relative to the direction of the step edges of the Ag(35 1 1) surface are illustrated in Figure 1. Because of the interactions at the step edges, only the chains A and C, as well as the chains B and D are energetically equivalent because of a remaining mirror plane perpendicular to the steps (see Figure 1). This is in contrast to the ideal (100) surface, where all four orientations are equivalent because of the additional rotational symmetry.

**Figure 2:**
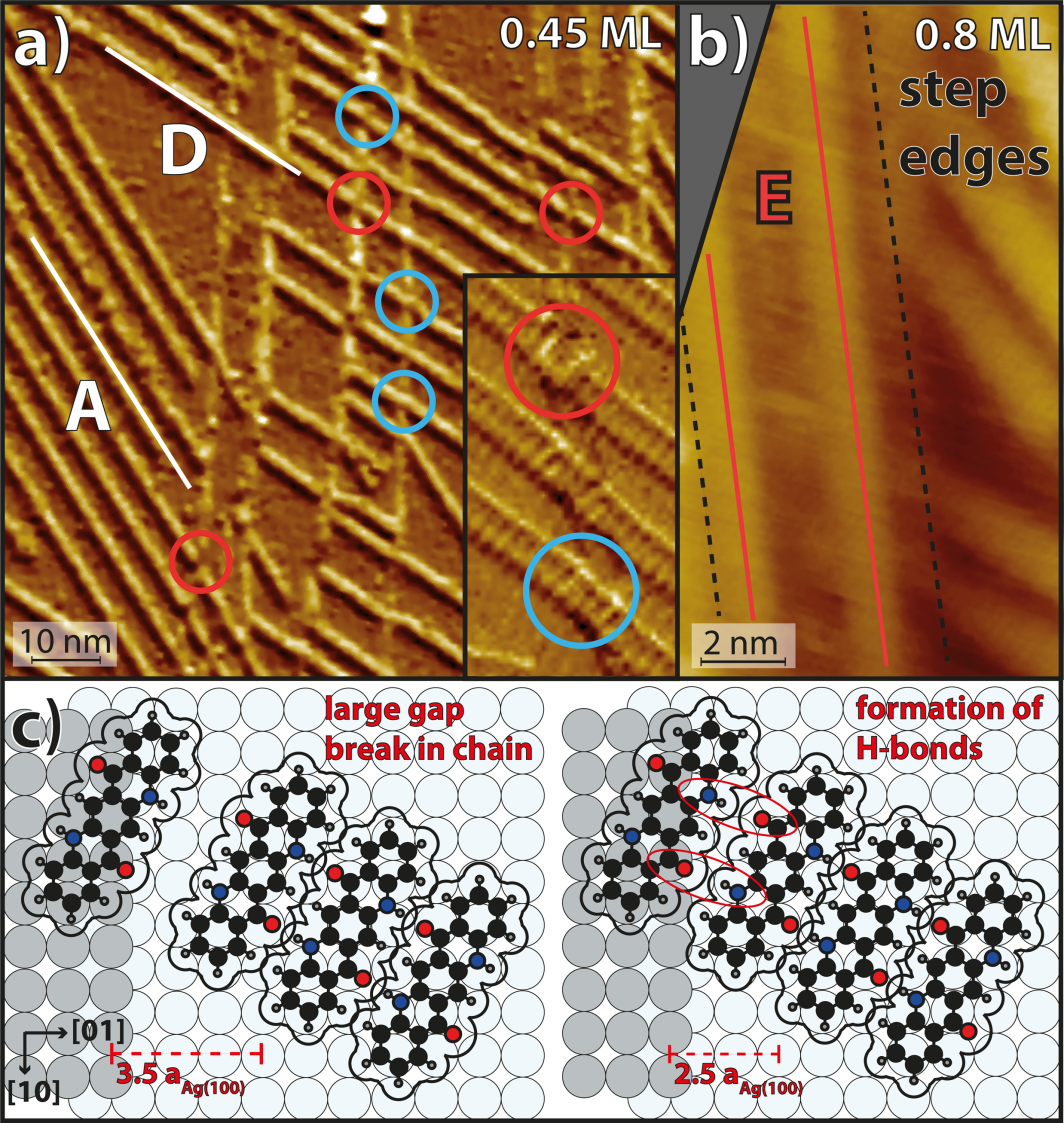
QA on the Ag(35 1 1) surface deposited at a sample temperature of 300 K. (a) STM image (*U*_Bias_ = −1.55 V, *I* = 40 pA) showing many parallel QA chains with the two azimuthal orientations A and D. The left-hand side of a step edge corresponds to its upper terrace and the right-hand side to its lower terrace. The red and blue circles highlight cases in which there is a break in a chain at a step edge and where the chains appears to grow seamlessly over the step edge, respectively. The inset shows an image at a different sample position of three QA chains at a Ag step edge, which was chosen for the high molecular resolution. (b) Small-scale STM image (*U*_Bias_ = 1.5 V, *I* = 25 pA) showing two of the four azimuthal orientations A and D, as well as two chains in the new orientation E (red lines). The black dashed lines indicate the positions of the step edges. (c) Models of chain growth at Ag step edges. These illustrate that the chains that nucleated on the lower terrace may statistically end in two positions with respect to the step edge. The left-hand side shows the case where the gap between the molecules on the upper and lower terrace is too large to allow for the formation of H-bonds. The right-hand side shows the case where the chain is one Ag lattice constant closer to the step edge, which allows for the formation of H-bonds across the step.

**Figure 3:**
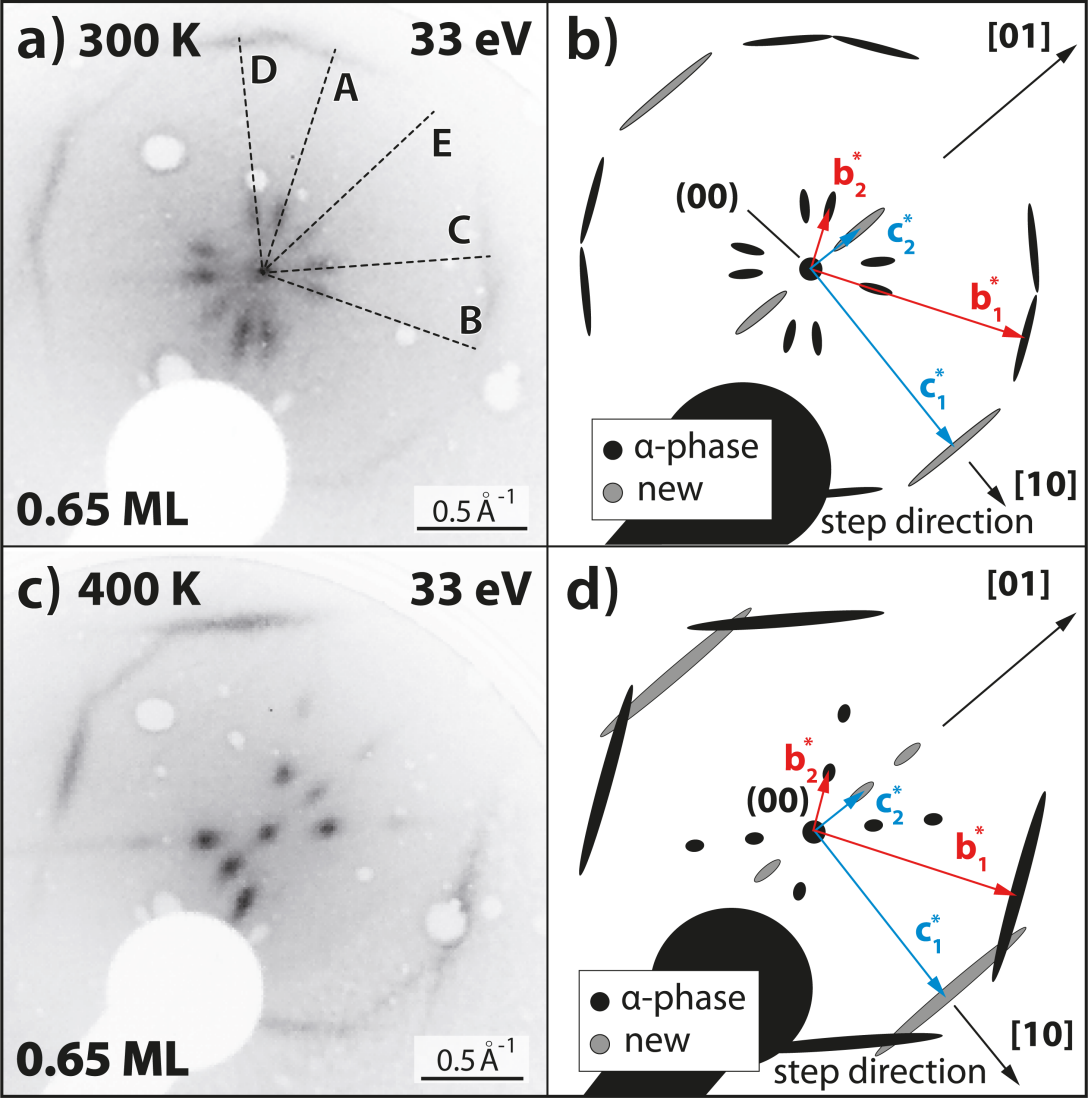
(a) LEED image (*E* = 33 eV, *T* = 300 K) of the region around the specular spot of 0.65 ML QA on Ag(35 1 1) after deposition at a sample temperature of 300 K. Note that the sample was rotated with respect to the normal incidence geometry. The diffraction pattern is very similar to the one of the QA α-phase on Ag(100) but contains additional spots that correspond to the new chain orientation E. The black dashed lines indicate the five azimuthal chain orientations. (b) Schematic drawing of the LEED pattern in panel (a). It shows the observed spots and the unit cell vectors of orientations A and E. (c) Same as (a) but for θ_QA_ = 0.65 ML and a deposition at 400 K. The diffraction pattern is very similar to the one in panel (a). However, it is missing two sets of spots corresponding to orientations B and D. (d) Schematic drawing of the observed LEED pattern in panel (c) containing all the observed spots and the corresponding unit cells. Both LEED images were measured under an in-phase condition for the specular spot with respect to monoatomic Ag steps [38]. This causes that the spots are sharp and not broadened because of the Ag steps. The scale bars for the LEED images are only approximately valid because of the distortion caused by the MCP geometry.

An STM image of the QA chains on Ag(35 1 1) is displayed in Figure 2a. It shows domains of parallel chains belonging to one of the azimuthal orientations A and D. This is an example of a common observation we made: In most areas of the surface with unidirectional step edges of not too small distance, these two out of the four orientations A–D were preferentially observed. However, we do not deduce yet that the orientations A and D are generally preferred over B and C because the statistics acquired from STM images are insufficient for reliable conclusions, although the images were deliberately collected from different regions of the sample. A corresponding LEED image is displayed below in Figure 3a and shows spots corresponding to the four azimuthal orientations with similar intensities. This shows that, on average, all four orientations are present on the surface with equal probability.

#### Growth across steps

A particularly striking observation from the STM image in Figure 2a is that, at step edges, the QA chains on the lower terrace very often continue at the exact position of the step edge where a chain on the upper terrace ends. For a coverage of 0.8 ML, this occurs in about 90% of all cases when a QA chain reaches a Ag step edge. At lower coverages, this phenomenon is less likely (at 0.45 ML only about 70%). When QA chains continue across the step, in about 50% of all cases a visible gap or break in the chain (discontinuity) can be seen (red circles in Figure 2a), while in the other 50% of all cases, it looks as if the chain grows undistorted across the Ag step (light blue circles). For a closer look, a zoom-in onto chains at Ag step edges with molecular resolution is displayed in the inset. The blue circle highlights a chain that grows across the step edge with all individual molecules being distinguishable. The red circle shows a chain with a discontinuity in the periodic structure, leading to a lateral offset between the two parts of the chain at the step edge. The chain in the middle of the inset (not marked by a circle) grows continuously across the Ag step, but three molecules close to the step edge appear slightly rotated with respect to the other molecules of the chain. This is an example of a slight distortion of the azimuthal orientations of the molecules in the chain, which we have observed frequently for chains growing continuously across Ag steps. In the literature, it has also been reported that linear molecules can bend when crossing a step edge [35–36]. We suppose that this is not the case here because the QA molecule is shorter and stiffer because of its planar structure. The above observation shows that the intermolecular H-bonds are capable of stabilizing the growth of QA chains across Ag step edges. The reason why the chains grow only occasionally continuously across the step edges will be discussed below. A similar observation of molecular chains that are formed by H-bonds and grow across step edges was made for 2,6-naphthalene-dicarboxylic acid (NDCA) on a vicinal Ag(110) surface [37].

From the above observations, conclusions about the chain growth can be drawn. The fact that the four azimuthal chain orientations A–D are present on the surface with equal probability indicates that, at 300 K, the nuclei of the chains start growing on the terraces where all four orientations are energetically equivalent, and not at the steps. The chain nuclei grow at both of their ends until both step edges of a terrace are reached. For an explanation of the continuous or distorted growth of the chains across the steps, we propose the following model: We note that, because of the specific orientation and size of the molecules, the distance between the ends of the chains and the step edge can vary by one Ag(100) lattice constant (*a*_Ag(100)_ = 2.89 Å [31]). This is illustrated by the two models in Figure 2c. We suppose that the adsorption of a QA molecule at the step edge on the upper/lower terrace induces a local change in the electron density at the step edge. This favors a specific adsorption site of a second QA molecule with the same chirality and azimuthal orientation at the step edge on the lower/upper terrace. Hence, the growth of the QA chain continues at a nearest position of the step edge where the chain on the upper/lower terrace ends. If both molecules come close enough to the step edge (see the two cases in Figure 2c), intermolecular H-bonds may form across the step, and the chain continues in an undistorted structure. In the alternative case, a small gap between the two molecules close to the step is formed.

#### New step-induced orientation

Another interesting observation is that, on Ag(35 1 1), a fifth azimuthal orientation exists, which is parallel to the Ag step edges. This orientation will be labeled E from now on. It does not occur on the nominally flat Ag(100) surface. This indicates that, on large terraces, the orientation E is less favorable than A–D, but it is stabilized by the step edges of the vicinal surface. A model of a chain with orientation E is illustrated in Figure 1a. Two chains of this orientation E on one terrace are highlighted by red lines in the STM image in Figure 2b. These kinds of chains were observed rarely by STM and were, in particular, never observed by STM on terraces wider than 50 Å. Nevertheless, a LEED image (discussed below) shows additional spots that need to be explained by this chain orientation. The LEED image indicates that even domains of several parallel chains (at least five or more) of orientation E are present. This difference between the STM and LEED data is again attributed to the fact that the STM images sampled only a limited region on the sample where such domains were not present. Furthermore, the fact that the chains of orientation E are parallel and close to the Ag step edges (as seen in Figure 2b) might have caused that they are difficult to image by STM and were, hence, overlooked.

We now discuss the corresponding features in the LEED images in detail. A LEED image of QA with a coverage of θ_QA_ = 0.65 ML is illustrated in Figure 3a. It is very similar to that of the α-phase of QA/Ag(100) but contains additional spots. The α-phase is defined as the phase of parallel QA chains at a coverage of θ_QA_ = 1.0 ML, at which the distance *b*_2_ between the chains is minimal [23]. The model of the diffraction pattern in Figure 3b shows the spots of the known α-phase diffraction pattern in black and the new spots in gray. The reciprocal vectors of the α-phase and those of the new orientation E are depicted in red and light blue, respectively. The spot described by the vector 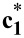 of orientation E corresponds to the intermolecular distances within the chains. This spot is also oriented in the [10] direction of the substrate, which confirms that the chain orientation E is indeed parallel to the Ag step edges. The other new spot, which is described by the vector 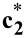, corresponds to the distance between neighboring QA chains in orientation E. The length 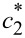 is identical to 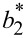, which means that the distance *c*_2_ between the chains of orientation E is identical to the distance *b*_2_ between neighboring chains of domains of the other orientations. This distance is *c*_2_ = *b*_2_ = 25 ± 2 Å and, hence, amounts to roughly half of the average terrace width (for the definition of **c_1_** and **c_2_** see Figure 1). This implies that only two chains of orientation E would fit onto one average terrace at this coverage. But from the sharpness of the spots, we conclude that these stem from domains of several parallel chains of orientation E, which have formed either on large terraces or even extend across the steps over several neighboring terraces. However, the spots given by the vector 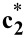 are still more smeared out (broader by 25%) than those from the α-phase, given by 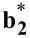. This can be explained by the fact that the domains of orientation E in the direction perpendicular to the chains are smaller than those of the other four orientations A–D.

The formation of similar one-dimensional structures parallel to the step edges of a vicinal crystal has been observed before, for example, PTCDA on Au(433) and Au(778) [16], a 1:1 mixture of PTCDI and 1,4-bis(2,4-diamino-1,3,5-triazine)benzene on Au(11 11 12) [17], α-6T on Ag(441) [39], and nickel tetraphenylporphyrin on Au (788) [18].

### Deposition at 400 K

Interestingly, after the deposition of QA onto Ag(35 1 1) at an elevated sample temperature of 400 K, the resulting QA structures have changed significantly. The LEED images (Figure 3c,d) show that the layer still consists of domains of parallel chains, but only the orientations A, C, and E are present. Moreover, the spots are smaller, sharper, and more intense than their counterparts in Figure 3a. This shows that the structures are less disordered and the domains (in the direction perpendicular to the chains) are larger than the ones after deposition at 300 K.

**Figure 4:**
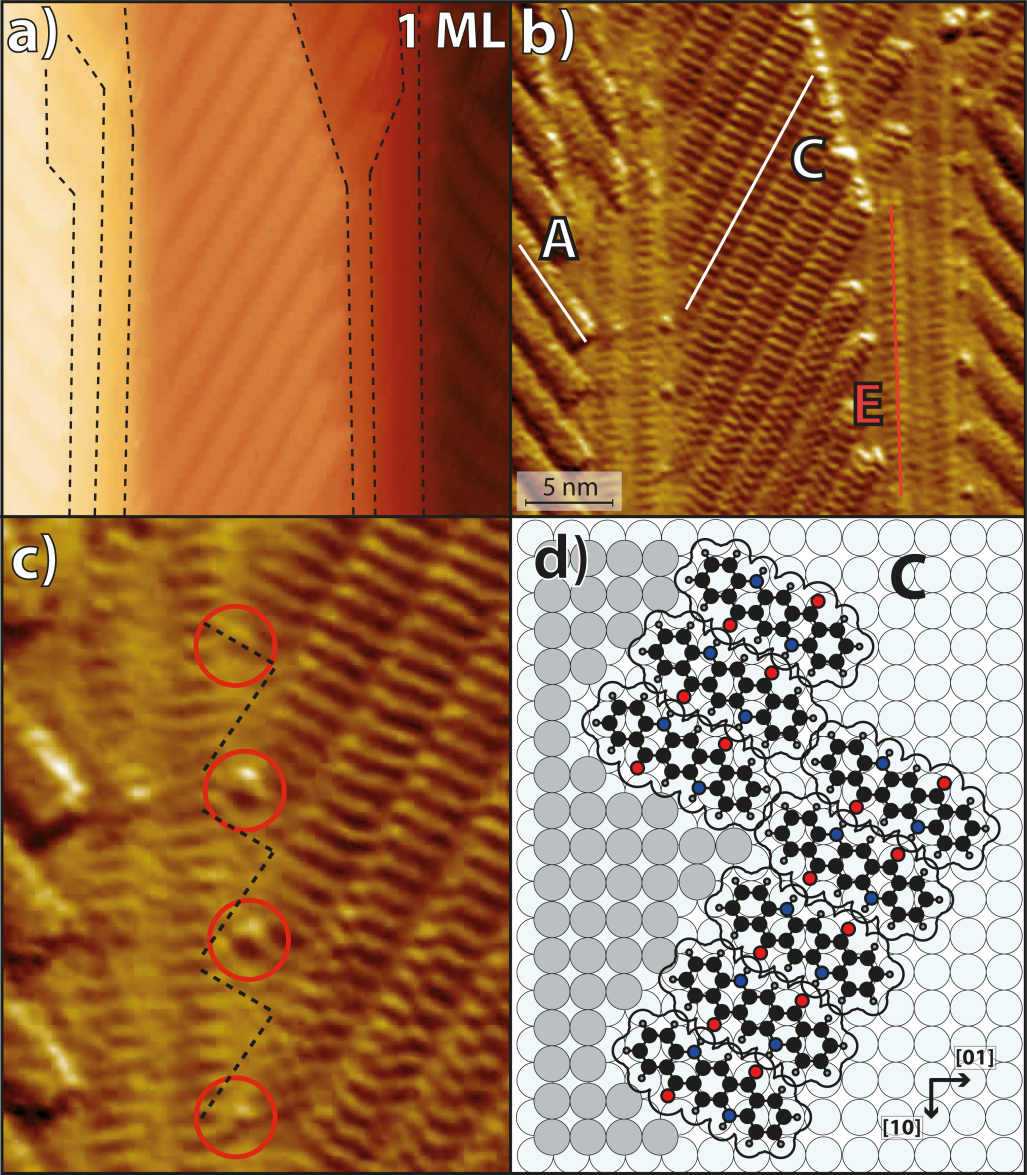
(a) STM image (*U*_Bias_ = −1.5 V, *I* = 25 pA) of QA on the Ag(35 1 1) surface deposited at a sample temperature of 400 K. It shows an area with several step edges running vertically through the image, which are highlighted by the dashed black lines. (b) The same STM image as in panel (a) with a Prewitt filter for better visibility of the QA structures. The image shows domains of parallel QA chains with the three orientations A, C, and E. (c) Zoom-in onto the STM image in panel (b) showing the QA chains at step edges. (d) Structure model of chains in orientation C at a step corresponding to panel (c). The black dashed lines mark the Ag step edges and the red circles highlight small circular protrusions that are assigned to small Ag clusters.

Corresponding STM images of different resolutions and sizes and a corresponding structure model are displayed in Figure 4. They show domains of parallel chains in the orientations A and C, as well as in the orientation E, which is parallel to the Ag step edges. The intermolecular distance within the chains remained unchanged for all azimuthal orientations at 6.6 ± 0.2 Å. The biggest difference from the structures after deposition at 300 K is the absence of the orientations B and D, which confirms above LEED results. This symmetry break can only be explained by the step edges and is a strong indication that, at 400 K, the nucleation of the chains proceeds now from the Ag step edges. The molecules in chains of orientations A and C (which exhibit equivalent adsorption geometries at the Ag step edges, see Figure 1) adapt more favorable adsorption sites than those in orientations B and D. Hence, the nuclei of orientations A and C are more stable. In addition, it may be possible that the increased mobility of the QA molecules causes short chains of orientations B and D to dissolve and integrate into the step-stabilized chains with the orientations A and C, instead.

A potential explanation for the higher stability of nuclei of orientations A and C may be that, at 400 K, Ag atoms can thermally detach from the Ag step edges. This enables the QA molecules to find a thermodynamically more stable adsorption geometry at the step edges in orientations A and C, possibly including a replacement of Ag atoms in the step edges by a part of the molecule. Indeed, a closer look at the STM image (see Figure 4c) shows a periodic zig-zag pattern of the Ag step edges (indicated by the black dashed lines). This is induced by the starting points of QA chains that replace some of the Ag atoms in the step edge in order to maximize the interactions between the molecules and the step edge. This is similar to the situation of PTCDA molecules at KCl, NaCl, or KBr step edges [40–42]. A corresponding structure model of QA chains at Ag step edges is displayed in Figure 4d. Furthermore, the STM image shows several small protrusions where the QA chains end at the Ag step edge. We assign these to small clusters of the Ag atoms that were expelled from the step edges by the QA molecules.

Another interesting difference from the structures after deposition at 300 K is that, after the deposition at 400 K, there are more chains with orientation E parallel to the step edges (highlighted by the red line in Figure 4b). Most small terraces (width *<* 50 Å) inspected by STM had at least one chain with orientation E close to the upward-pointing step edge. The higher abundance of orientation E after deposition at 400 K is also confirmed by LEED because the corresponding spot is more intense and sharper (see Figure 3). The growth of chains in orientation E parallel to the step edges is favored at elevated temperatures because, for these chains, the intermolecular H-bonds are not distorted by the steps. In addition, the chains are stabilized by the long-range dispersion interactions of the QA with the atoms of the step edge.

A further observation is that, after deposition at 400 K, no cases of chains continuously growing across Ag step edges could be observed anymore. The reason is that now the growth of the chains begins at the step edges, and the QA molecules at the start of the chains exhibit a strong bond to the Ag atoms of the step edges. Hence, they cannot provide H-bonds for chain growth across the steps. A second reason is that now the orientation E is significantly more prevalent than after deposition at 300 K, and these chains block other chains from growing across the steps.

Overall, these results show that the higher temperature of 400 K has led to the preferred formation of the three chain orientations A, C, and E, which are thermodynamically more stable than the orientations B and D.

### Deposition at 500 K

Last, we discuss the structure of QA after deposition at 500 K. From our previous work regarding QA on Ag(100), we know that the elevated temperature induces an irreversible phase transition into a heterochiral and commensurate structure, the so-called β-phase [23]. This phase is also obtained after deposition at 500 K and subsequent cooling to 300 K. This preparation was chosen here. The β-phase also consists of parallel molecular chains of QA dimers as the α-phase, but with periodic indents along the chains. The molecules in the domains of the β-phase exhibit the same azimuthal orientations as those in the α-phase. We find that the β-phase also forms on Ag(35 1 1), and the domains of the β-phase on Ag(35 1 1) will also be labeled A–D.

**Figure 5:**
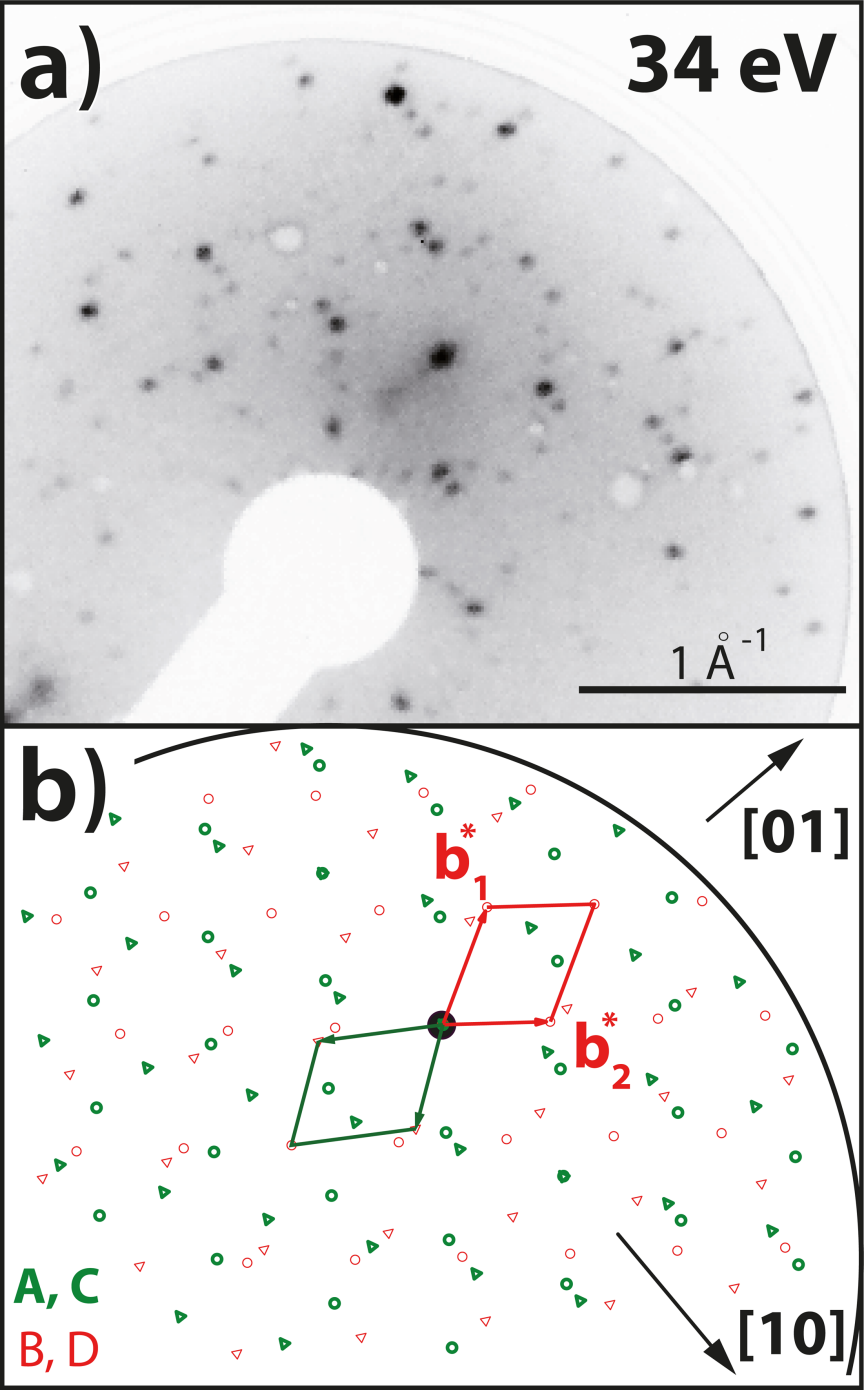
(a) LEED image (*E* = 33 eV, *T* = 300 K) of the β-phase of 0.65 ML QA on Ag(35 1 1) after deposition at a sample temperature of 500 K and subsequent cooling to 300 K. The diffraction pattern is very similar to the one of the QA β-phase on Ag(100) but exhibits a clear anisotropy. Roughly half of the spots are more intense than the other half. This LEED image was measured under an in-phase condition for the specular spot with respect to monoatomic Ag steps. (b) Simulated LEED pattern of the β-phase. The spots that are thicker and green correspond to the more intense spots in the diffraction pattern, which correspond to orientations A and C. The step direction is in the [10] direction, as before.

A corresponding LEED image (θ_QA_ = 0.65 ML) and a simulation are displayed in Figure 5a and Figure 5b, respectively. The spot positions in the LEED image are identical to those of the LEED image of QA on Ag(100) and, in principle, all four domains are seen. However, a striking difference is that, here, there is a very prominent inequivalence regarding the spot intensities. Half of the spots have significantly less intensity than the other half, which means that two domains of the β-phase are more prevalent than the other two domains. These are the domains A and C. This shows that, at 500 K, the growth of the β-phase also begins at the step edges, which leads to the observed preferential growth of two domains. This is in agreement with the above results regarding the growth of QA at 400 K and supports the scenario that the growth of QA chains starts at the Ag step edges at elevated temperatures.

All spots of the LEED pattern are very sharp. Hence, the corresponding domains are significantly larger than the average terrace width of 50 Å. A coherent continuation of the domains across the Ag step is very unlikely. As stated above, the terrace width distribution of the Ag(35 1 1) surface is rather broad with terrace widths of up to 300 Å. Thus, we conclude that the β-phase predominantly forms on those large terraces. This is also supported by the STM results, which show that large terraces were completely covered by one of the four β-phase domains (not shown). Furthermore, in areas of the sample where many step edges are very close to each other and the terraces are very small (below 50 Å), no QA molecules were observed on the terraces. This indicates that the molecules diffused off the small terraces onto larger ones because the space on the small terraces is not sufficient to form the β-phase. One may also ask whether this preference of the β-phase for large terraces leads to a step bunching on the substrate surface in order to increase the size of the terraces. However, we could not find any evidence that the terrace width distribution changed significantly after the preparation of the β-phase.

As already postulated above, we assume that, upon adsorption at the step edges, the QA molecules replace Ag atoms in order to maximize their adsorption energy. The nuclei of orientations A and C are more stable; thus, the growth of the corresponding domains is favored compared to that of the orientations B and D. Nevertheless, the orientations B and D are still present on the surface to some minor extent, which can possibly be explained by domains that nucleate and grow on large terraces and not at steps.

## Final Discussion

### Energetic considerations regarding chains parallel to the steps

The chain orientation E is not present on the nominally flat Ag(100) surface [23], which means that it must be induced by the step edges of the vicinal surface. On Ag(35 1 1), chains of orientations E were mainly observed close to step edges, that is, the far end of the molecule was within 20 Å. Remarkably, we do not find any step decorations by the QA molecules with their long axis close and parallel to the steps. Nevertheless, the step edges play an important role in the formation of chains of orientation E. The absence of chains with orientation E on the nominally flat surface shows that orientation E is energetically less favorable than the orientations A–D in terms of direct interactions between the QA molecules and the Ag(100) surface. This means that the nuclei with orientation E are less stable than those with orientations A–D on terraces. However, if chains of orientation E grow parallel to step edges, they are additionally stabilized because, owing to the close proximity, every molecule in the chain is subject to attractive dispersion interactions with the Ag step edge. This is not the case for orientations A–D, which grow away from the step edges. A model of such a chain of orientation E at a step edge is illustrated in Figure 1a.

A second aspect that favors the growth of chains with orientation E on small terraces is that, because they are parallel to the step edges, they can grow without encountering any distortions at Ag steps, as is the case for long chains of the orientations A–D in areas with a high density of unidirectional steps. Nevertheless, on the small terraces, the formation of chains with orientation E may be kinetically inhibited because of a lack of space, which hinders the diffusion of the molecules to the growing endpoints of the chains. This is supported by the fact that an increased mobility of QA molecules (i.e., during deposition at a sample temperature of 400 K instead of 300 K) promotes the formation of chains with orientation E.

### QA chain growth across Ag step edges

As stated above, at 300 K, the QA chains are capable of growing continuously across Ag step edges. This brings forth some interesting aspects, which we discuss here. A chain across step edges without a significant break and intact intermolecular H-bonds requires some structural flexibility in the molecules and/or the intermolecular bonds. We suppose that QA molecules cannot bend in the plane of the π systems because of the annulated carbon rings. In contrast, H-bonds are mainly of electrostatic nature and, thus, have a pronounced flexibility in bond lengths and angles, which supports intermolecular bonds across step edges. Similar molecular chains that are connected by H-bonds and grow across monoatomic step edges without a break were observed before for NDCA on a vicinal Ag(110) surface by Schnadt et al. [37]. They found that the slight distortion of the chains at step edges leads only to a minor energy loss (below 0.1 eV), which is energetically still more favorable than a scenario in which no H-bonds are formed across the step edges. We propose that a similar situation is present here.

Another interesting question concerns the angle between the QA chains and the step edges. Schnadt et al. found that the self-assembly across step edges crucially depends on the azimuthal orientations of the chains relative to the step edges [37]. The chains of NDCA grow along the [10] direction of the substrate and are, thus, orthogonal to the direction of the step edges, which are oriented along the [01] direction. Density functional theory calculations have shown that the NDCA chains do not cross the step edges if they are rotated away from the close-packed rows [37]. In contrast, in our case, no chains grow across the step edges at a 90° angle. The QA chains continuously grow across the step edges in the four azimuthal orientations A–D, resulting in angles of 32° and 58° between the chains and the Ag step edges. Hence, such a strict selection of the azimuthal angle of the chains, similar to the one found by Schnadt et al., does not apply to QA.

### Anisotropic β-phase

Now we discuss an interesting aspect regarding the β-phase on Ag(35 1 1), namely the fact that the minority orientations B and D are present on the surface after the deposition at 500 K, although chains with these orientations could not be observed after the deposition at 400 K (see Figure 3c and Figure 4). This can be explained by the stability of the different QA phases. After the deposition at 500 K and subsequent cooling, the β-phase domains of orientations B and D have formed on the large terraces in addition to the majority domains with orientations A and C. The transition from a disordered phase after deposition at 500 K to the ordered β-phase occurs at about 450 K. These domains are more stable than the single chains with the same orientations B and D of the α-phase because of their commensurate nature. Hence, they do not completely transform into the domains of orientations A and C.

One might also ask whether annealing the sample at 500 K for a longer time, or several consecutive annealing steps at 500 K, might further change the ratio in favor of orientations A and C. This was not the case. We observed the same intensity ratio between the stronger and weaker spots in the LEED pattern after every preparation and several consecutive annealing cycles. This can be explained by the fact that, at 500 K, the β-phase again dissolves into a disordered phase and only forms again upon cooling [23]. Hence, the cooling rate may be relevant for the ratio between orientations A/C and B/D in the β-phase, but this was not investigated, yet.

### Comparison to literature

Last, we discuss some similarities and differences between our results and some that were published previously. One similarity to other molecular systems is the growth of the QA chains with orientation E, which grow along the Ag step edges. Such a behavior was found for a few other organic molecules on different vicinal metal surfaces [17–18]. This shows that the contribution of the interactions between the atoms of the step edges and the organic molecules to the adsorption energy of the molecules is a common and important motif for the adsorption and self-assembly of organic molecules on stepped surfaces. We note, however, that the orientations of the QA molecules with respect to the steps at 300 K are unusual. Many examples from the literature that report on the decoration of steps by organic molecules found that the molecules adsorb in a flat-lying configuration with their long axis parallel to the step edges [43–45]. In some cases, an adsorption of upright standing molecules at steps was also found [45–46]. Here, the QA molecules are either oriented with their short edge parallel to the steps or at an angle of 32° or 58° with respect to the step direction. A stark difference to the examples described in the above publications is that, here, the Ag step edges also cause the preferential growth of QA chains or commensurate domains with distinct step-selected orientations at elevated temperatures. A similar phenomenon has, to the best of our knowledge, not been reported, so far.

Another phenomenon that was reported by Schmitt et al. [47] for PTCDA on vicinal Ag(100) surfaces is the faceting of the Ag substrate surface that is induced by the adsorption of the molecules. They found that the adsorption of PTCDA leads to the formation of Ag facets, which are usually not stable on the bare surface and are induced by the adsorption of PTCDA. However, within the scope of the present work, we did not observe any faceting of the Ag(35 1 1) surface. This is because the adsorption energy of QA is likely significantly smaller than the one of PTCDA on Ag surfaces.

## Conclusion

We found that, at room temperature (RT), the growth of QA on the vicinal Ag(35 1 1) surface is, in general, very similar to its growth on the nominally flat Ag(100) surface. QA forms molecular homochiral chains with four distinct symmetry-equivalent orientations that are connected via H-bonds (α-phase). At RT, the growth of the chains begins on the Ag terraces, and the unidirectional step edges do not have any influence on the azimuthal orientations of the chains. Furthermore, the QA chains grow across the steps. We also found one azimuthal chain direction that does not exist on Ag(100) and is parallel to the unidirectional step edges of the vicinal surface. This is explained by the fact that the growth in this direction is additionally stabilized by the dispersion interactions between the QA molecules and the atoms of the Ag step edges, which overcompensates the lower site energy of the molecules on the terraces in this orientation.

After deposition at 400 K, only two of the above four azimuthal orientations are observed. Furthermore, the deposition at 500 K leads to heterochiral chains in a commensurate structure (β-phase) that also exhibits a strong anisotropy, that is, two domains are favored over the other two. In both situations, the preferential growth of specific orientations at 400 and 500 K is caused by a growth start of the QA structures at the Ag step edges at elevated temperatures. Thus, the step edges have a strong influence on the azimuthal orientations of the QA structures because adsorption sites at the Ag step edges of molecules in two distinct azimuthal orientations out of four are favored.

Overall, the azimuthal orientations of one-dimensional QA chains can be influenced by step edges on vicinal surfaces. Our work is of importance for the general understanding of the interactions between organic molecules and metallic step edges and may be helpful for the preparation of one-dimensional organic structures with a global preferential orientation on surfaces.

## Supporting Information

The Supporting Information features an STM image of the bare Ag(35 1 1) surface and a corresponding step edge distribution.

File 1 Terrace width distribution on Ag(35 1 1)

## Data Availability

The data that supports the findings of this study is available from the corresponding author upon reasonable request.
