# Supplementary material for: Directed growth of quinacridone chains on the vicinal Ag(35 1 1) surface
Source: Beilstein J Nanotechnol. 2024 May 21;15:556–68. doi: 10.3762/bjnano.15.48 (PMC11181275; doi:10.3762/bjnano.15.48)
Supplement: File 1 — Terrace width distribution on Ag(35 1 1) [file Beilstein_J_Nanotechnol-15-556-s001.pdf]

BEILSTEIN JOURNAL OF NANOTECHNOLOGY

Supporting Information

for

Directed growth of quinacridone chains on the vicinal Ag(35 1 1) surface

Niklas Humberg, Lukas Grönwoldt and Moritz Sokolowski

Beilstein J. Nanotechnol. **2024,** *15,* 556–568. doi:10.3762/bjnano.15.48

Terrace width distribution on Ag(35 1 1)

License and Terms: This is a supporting information file under the terms of the Creative Commons Attribution License (https://creativecommons.org/licenses/by/4.0). Please note that the reuse, redistribution and reproduction in particular requires that the author(s) and source are credited and that individual graphics may be subject to special legal provisions.
The license is subject to the *Beilstein Journal of Nanotechnology* terms and conditions: (https://www.beilstein-journals.org/bjnano/terms)

In Figure S1a, a typical STM image of the clean Ag(35 1 1) surface is shown. It can be seen that the step edges are not evenly distributed with the nominal terrace width of 50.5 Å. Instead, there are areas on the surface where many step edges are very close together. For example, on the left-hand side of the image, there are six step edges within a distance of roughly 120 Å. This means that the average terrace width in this area is only 20 Å, which is less than half of the terrace width of the ideal Ag(35 1 1) surface. At the same time, there are also very large terraces on the surface. In Figure S1, the large terrace is 270 Å in width, which is more than five times of the ideal terrace width.

The terrace width distribution is displayed in Figure S1b. It shows a dominant peak at around $n = 10$ atoms rows (= 28.9 Å). For larger terrace widths, it follows a decay that can be roughly described by a geometric distribution, which is indicative of non-interacting steps [1]. However, for small terrace widths (less than ten rows) it exhibits a drastic decline, which shows that there is a repulsive interaction between the steps for very small terrace widths.

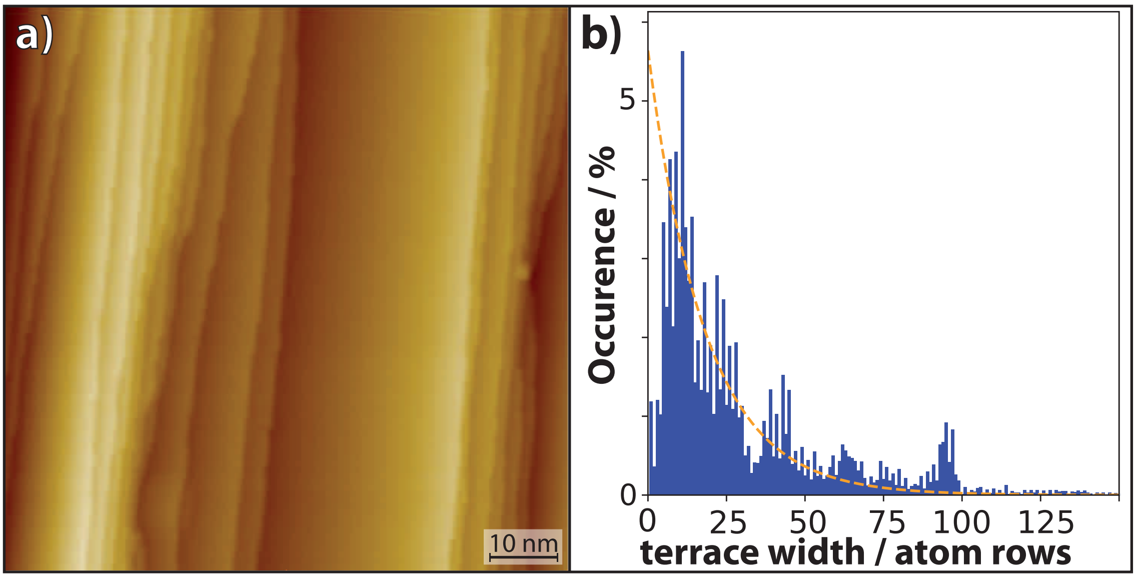

Figure S1: Step morphology of the Ag(35 1 1) surface. **(a)** STM image (U_{Bias} = +1.5 V, I = 25 pA). **(b)** Terrace width distribution. It was obtained by evaluating four large-scale STM images with a Python script by Bastidas et al. [2]. The local peaks at $n > 25$ are due to the fact that, because of the finite number of evaluated STM images, individual large terraces are oversampled by the script because it analyzes every horizontal line of the image. The orange dashed line corresponds to a fit by a geometric distribution, that is, occurrence $\propto p^n$, with n denoting the number of rows per terrace and p being the probability for two adjacent atomic rows at the same height level.

References

1. Busch, H.; Henzler, M. *Surf. Sci.* **1986**, *167*, 534–548. doi:10.1016/0039-6028(86)90722-3.

2. Bastidas, J. M. P.; Auras, S. V.; Juurlink, L. B. *Appl. Surf. Sci.* **2021**, *567*, 150821. doi:10.1016/j.apsusc.2021.150821.
